# Chronic Obstructive Pulmonary Disease (COPD) is associated with pulmonary artery stiffness - the MESA COPD study

**DOI:** 10.1186/1532-429X-15-S1-O62

**Published:** 2013-01-30

**Authors:** Chia-Ying Liu, Megha Parikh, Antoinette S Gomes, Jens Vogel-Claussen, David A Bluemke, Joao A Lima, Martin R Prince, Graham Barr

**Affiliations:** 1 Johns Hopkins University, Baltimore, MD, USA; 2 Department of Epidemiology, Mailman School of Public Health, Columbia University, New York, NY, USA; 3 Department of Medicine, University of California, Los Angeles, Los Angeles, CA, USA; 4 Hannover Medical School, Hannover, Germany; 5 Radiology and Imaging Sciences, National Institutes of Health, Bethesda, MD, USA; 6 Department of Medicine, Weill Cornell Medical College, New York, NY, USA

## Background

This study seeks to evaluate indices of pulmonary artery (PA) stiffness in patients with COPD and compare with normal controls. We hypothesize that patients with COPD would have increased pulmonary artery stiffness. To test this we determine the pulmonary artery area change (distensibility in %) by cardiac MRI and relate the distensibility to a wide range of severity of COPD.

## Methods

The MESA COPD Study recruited 290 patients (135 patients of various COPD severity and 155 controls) from four field centers in the US, age 50-79 years with ≥10 pack-years of smoking, all free of clinical cardiovascular disease. COPD was defined on post-bronchodilator spirometry by GOLD criteria (FEV1/FVC <70%, FEV1 % predicted >80% = mild, 50-80%=moderate, <50%=severe). All participants underwent full-lung CTs. Percent emphysema was defined as the percentage of total voxels within the lung field that fell below -910 Hounsfield units. MRI studies were performed using 1.5T scanners. To measure ventricular function, the entire heart was imaged in short-axis orientation using a retrospectively gated steady-state free precession sequence. Phase-contrast images of the pulmonary arteries were obtained using a segmented fast gradient echo sequence with free breathing and analyzed quantitatively using dedicated software (FLOW, Medis). Distensibility of the pulmonary vessels (in %) are measured by the following formula, 100×(maximum PA area-minimum PA area)/minimum PA area. The base model (model 1) was adjusted for age, gender, height, weight, race/ethnicity and cohort of selection, given relationships of COPD severity to the pulmonary distensibility. We then additionally adjusted for smoking status, pack-years, diabetes mellitus, hypertension, oxygen saturation, LDL, HDL and statin use (model 2).

## Results

Table 1 summarizes the clinical characteristics of 290 participants stratified by COPD severity. Distensibility of the main, right and left PA was reduced in COPD compared to controls in both models (Table 2). Main and right pulmonary distensibilities were inversely related to percent emphysema after minimal adjustment (model 1, P=0.21 and 0.07, respectively) and similar trends with statistical significance in the full model (model 2, P=0.049 and 0.01, respectively). Pulmonary distensibilities was positively associated with the percent predicted FEV1 but only left PA attain statistical significance after base adjustment (model 1, P=0.047).

**Table 1:** Clinical characteristics of participants in the MESA COPD Study stratified by COPD severity.

	Controls (n=155)	COPD Mild(n=54)	COPD Moderate (n=65)	COPD Severe (n=16)
Age, mean±SD, years	67.95±6.55	70.15±6.25	67.80±7.36	68.75±5.90

Sex, male, No. (%)	83 (53.55)	41 (75.93)	38 (58.46)	10 (62.50)

Body mass index, mean±SD, kg/m2	28.66±4.96	26.63±3.98	27.29±5.09	27.56±6.21

Cigarette smoking status, Current, No. (%)	34 (21.94)	13 (24.07)	26 (40.00)	4 (25.00)

Hypertension, No. (%)	65 (41.94)	23 (42.59)	32 (49.69)	7 (43.75)

Diabetes Mellitus, No. (%)	24 (15.48)	5 (9.26)	9 (13.85)	4 (25.00)

FEV1 percent of predicted, mean±SD	101.26±15.64	92.15±9.61	68.58±8.29	39.56±7.54

Percent Emphysema-910, median	15.30	28.94	23.86	37.63

LV End Diastolic Mass, mean±SD, g	125.66±32.91	132.4±32.58	130.17±36.66	131.96±40.47

LV End Diastolic Volume, mean±SD, mL	120.38±31.00	120.61±28.47	114.84±30.46	102.80±42.93

LV End Systolic Volume, mean±SD, mL	47.09±17.94	47.17±15.73	47.23±17.65	42.85±21.11

LV Stroke Volume, mean±SD, mL	73.30±17.37	73.43±18.01	67.61±18.42	59.98±23.84

LV Ejection Fraction, mean±SD, %	61.50±6.84	61.20±7.55	59.17±7.71	59.24±7.04

LV Cardiac Output, mean±SD, L/min	5.03±1.29	4.86±32.58	4.76±1.27	4.45±1.50

**Table 2:** Mean difference (in %) and predicted mean levels (in %) of distensibility in the MESA COPD Study by COPD severity.

	Controls n=155	Mild n=54	Moderate n=65	Severe n=16	P-Trend
Distensibility of main PA					

Model 1, mean difference	Reference	-1.49	-1.44	-2.78	0.04

Model 1, predicted mean	15.41	13.91	13.97	12.67	

Model 2, mean difference	Reference	-1.48	-1.44	-2.56	0.08

Model 2, predicted mean	15.40	13.93	12.49	9.94	

	n=143	n=50	n=62	n=14	

Distensibility of right PA					

Model 1, mean difference	Reference	-0.58	-3.30*	-3.83	0.005

Model 1, predicted mean	20.59	20.01	16.71	12.88	

Model 2, mean difference	Reference	-0.93	-3.62*	-3.85	0.01

Model 2, predicted mean	20.67	19.74	16.12	12.27	

Distensibility of left PA					

Model 1, mean difference	Reference	-4.17*	-3.34*	-4.08	0.002

Model 1, predicted mean	18.26	14.08	10.74	6.67	

Model 2, mean difference	Reference	-4.63*	-3.33*	-2.66	0.003

Model 2, predicted mean	18.25	13.62	10.29	7.64	

					

*p < 0.05 Model 1 adjusted for age, race, gender, height, weight, and cohort Model 2 adjusted for variables in model 2 in addition to smoking status, pack years, educational attainment, diabetes mellitus, hypertension, oxygen saturation, LDL, HDL and statin use.

## Conclusions

We conclude that in COPD patients without overt cardiovascular disease, pulmonary artery distensibility is reduced. Higher pulmonary arterial stiffness also correlated with the percent emphysema on CT scan and FEV1.

## Funding

National Institutes of Health R01-HL093081 R01-HL077612, R01-HL075476 and N01-HC95159-HC95169, UL1 RR024156.

